# A data-based study in support of Blackbuck related cases from Haryana

**DOI:** 10.1016/j.dib.2018.02.034

**Published:** 2018-02-16

**Authors:** Vikas Kumar, Neelkamal Sharma, Arun Sharma, Kapil Verma, Kusum Singal, Mahesh Kumar

**Affiliations:** a Department of Genetics, M.D. University, Rohtak 124001, Haryana, India; b Directorate of Forensic Science, Himachal Pradesh, Shimla Hills, Junga (India)

**Keywords:** Blackbuck, Wildlife, Conservation

## Abstract

Haryana State (located in the northern part of India), is lacking in natural forest, but it has rich biodiversity of some wild animals, especially the Blackbuck antelopes. The maximum population of Blackbucks in the state is living in open cultivated fields. Blackbucks were once found abundantly throughout Haryana, but now they are limited to the south–west part of the state, driven almost to extinction in the rest of Haryana, mainly because of habitat destruction and wildlife crime. This data report is an outcome of six years (January 2012–September 2017), based on assessment of records in terms of location, year and month wise frequency of death and rescued cases related to Blackbuck reported, as registered by the State Wildlife Department. It is envisioned that this data report will provide appropriate information for the conservationist to plan future conservation strategy for Blackbucks in Haryana.

**Specifications table**

**Table:** 

Subject area	Wildlife Conservation
More specific subject area	Blackbuck Conservation
Type of data	Table, text file, graph, figure.
How data was acquired	Data has been acquired by survey method from Wildlife Department of Haryana, India.
Data format	Filtered, analyzed.
Experimental factors	Nil
Experimental features	The distribution patterns of death and rescued related data has been evaluated in terms of location, year and month wise and computed in the form of tables and graph.
Data source location	Haryana, India (27°39′ to 30°35′ N latitude and between 74°28′ and 77°36′ E longitude).
Data accessibility	Data is available with this article.

**Value of data**

• Very little is known about the current mortalities of Blackbucks in Haryana and there are few conservation data.
• On the basis of the data collection, this study demarcated Haryana into different zones based on population density and reported cases of Blackbuck (Fig. 1).

**Fig. 1:**
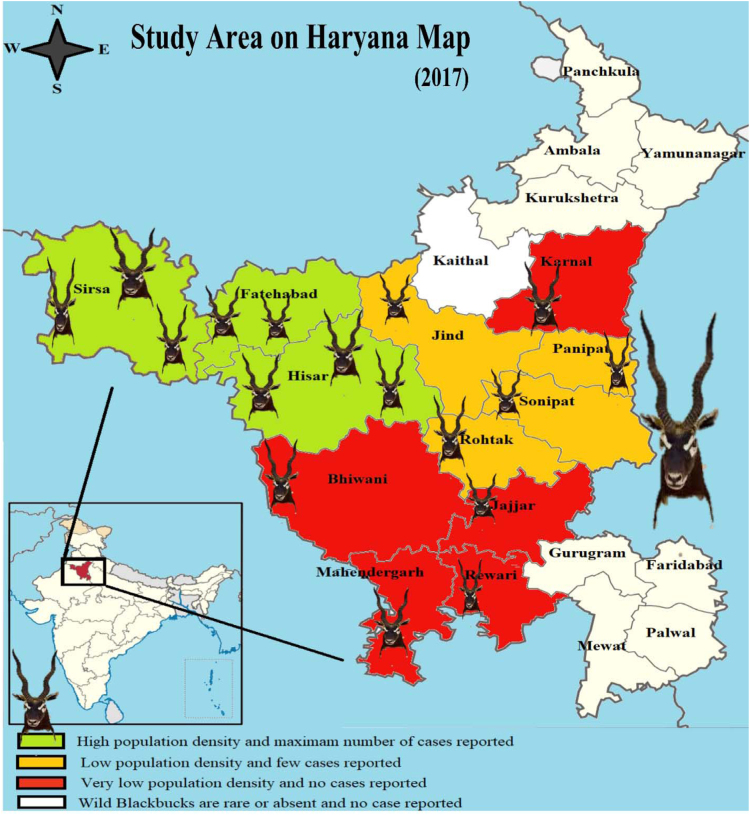
Map of Haryana showing study area. Different colors in map showing different zones demarcated on the basis of population density and reported cases.
• The data provided here is first of its kind related to Blackbuck from Haryana, will help to the readers to fully understand types and trends of Blackbucks death and their conservation efforts.
• Data can provide detailed information for wildlife conservationists to understand mortality trends and develop new strategies and policies for Blackbuck conservation.
• Such data are also important for researchers working in the fields of forensic science and environmental science, for the assessment of wildlife crime and environmental change.
• The data will provide a source of information for government organizations to develop an action strategy to protect the vulnerable wildlife of the state.

## 1 Data

The Blackbuck antelopes (*Antilope cervicapra*) are native to the Indian subcontinent, is the only living member of the genus Antilope [1]. The alarming rate of the population reduction of the Blackbuck is a matter of concern for the state. This research is an outcome of a reflective study based on assessment of records related to Blackbucks death and rescued Blackbucks cases reported from Haryana. The data in this study are limited to those cases which were registered by the State Wildlife department.

## 2 Experimental design, materials and methods

**Fig. 2:**
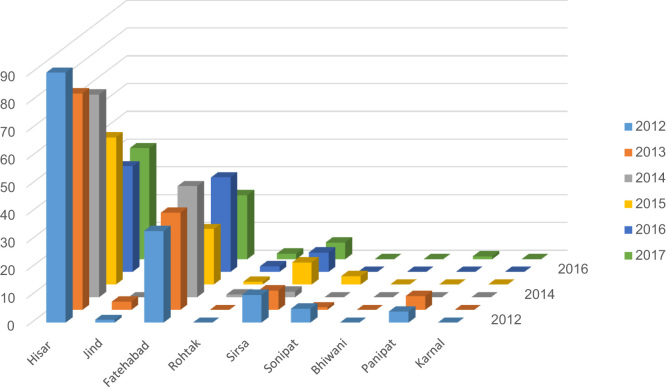
Year wise distribution of the Blackbucks death cases reported from different districts of Haryana.

The experimental design was based on collection of data on distribution patterns of deaths among Blackbucks and rescued Blackbucks reported in Haryana. Approximate last six years data from January 1, 2012 to September 30, 2017 were collected from the district headquarters of the Haryana Wildlife Department. The data were gathered in terms of district, year and month wise and have been reviewed and summarized in the form of tables (Table 1, Table 2, Table 3, Table 4) and graph (Fig. 2). Permission for this scientific research is granted by the National (MoEF&CC) and the State Wildlife department vide letters no 1–56/2016 and WL-87/11-04-16, respectively.

**Table 1:** District and year wise data of the Blackbucks death cases reported.

**District**	**Number of death cases reported**						**Total Cases**
	**2012**	**2013**	**2014**	**2015**	**2016**	**2017 (up to September)**	
**Hisar**	90	78	73	53	38	40	**372**
**Jind**	1	3	0	1	0	0	**5**
**Fatehabad**	33	35	40	20	34	23	**185**
**Rohtak**	0	0	1	1	2	2	**6**
**Sirsa**	10	7	2	8	7	6	**40**
**Sonipat**	5	1	0	3	0	0	**9**
**Bhiwani**	0	0	0	0	0	0	**0**
**Panipat**	4	5	0	0	0	1	**10**
**Karnal**	0	0	0	0	0	0	**0**
**Total**	**143**	**129**	**116**	**86**	**81**	**72**	**627**

**Table 2:** Distribution frequency of the Blackbucks death cases reported.

**Case type**	**Number of cases**	**Frequency of cases (%)**
**Road Accident cases**	24	3.82
**Feral dogs + fencing cases**	570	90.90
**Killed by Human**	8	1.27
**Natural death reported**	4	0.63
**Disease reported**	21	3.34

**Table 3:** Month and year wise data of reported death cases in Haryana.

**Years**	**2012**		**2013**		**2014**		**2015**		**2016**		**2017 (Up to September)**		**Total Cases (Death)**
**Month**	**No of cases**	**% of cases**	**No of cases**	**% of cases**	**No of cases**	**% of cases**	**No of cases**	**% of cases**	**No of cases**	**% of cases**	**No of cases**	**% of cases**	
**January**	17	11.8	26	20.1	11	9.4	14	16.2	7	8.6	14	19.4	**89**
**February**	11	7.6	10	7.7	14	12.0	3	3.4	5	6.1	9	12.5	**52**
**March**	7	4.8	9	6.9	3	2.5	4	4.6	4	4.9	9	12.5	**36**
**April**	8	5.5	6	4.6	7	6.0	10	11.6	9	11.1	7	9.7	**47**
**May**	15	10.4	17	13.1	17	14.6	11	12.7	9	11.1	14	19.4	**83**
**June**	9	6.2	14	10.8	9	7.7	9	10.4	4	4.9	9	12.5	**54**
**July**	14	9.7	15	11.6	7	6.0	8	9.3	13	16.0	6	8.3	**63**
**August**	11	7.6	9	6.9	6	5.1	1	1.1	6	7.4	3	4.1	**36**
**September**	8	5.5	4	3.1	8	6.8	6	6.9	3	3.7	1	1.3	**30**
**October**	8	5.5	8	6.2	5	4.3	8	9.3	10	12.3	__	__	**39**
**November**	23	16.0	6	4.6	16	13.7	5	5.8	4	4.9	__	__	**54**
**December**	12	8.3	5	3.8	13	11.2	7	8.1	7	8.6	__	__	**44**
**Total**	**143**		**129**		**116**		**86**		**81**		**72**		**627**

**Table 4:** Month and year wise data of the rescued cases in Haryana.

**Years**	**2012**		**2013**		**2014**		**2015**		**2016**		**2017 (Up to September)**		**Total Rescued Cases**
**Months**	**No of cases**	**% of cases**	**No of cases**	**% of cases**	**No of cases**	**% of cases**	**No of cases**	**% of cases**	**No of cases**	**% of cases**	**No of cases**	**% of cases**	
**January**	–	–	5	7.04	6	4.9	10	10.7	7	9.2	10	11.2	**38**
**February**	–	–	9	12.6	24	19.8	4	4.3	4	5.2	11	12.3	**52**
**March**	–	–	5	7.0	2	1.6	7	7.5	6	7.8	7	7.8	**27**
**April**	–	–	9	12.6	5	4.1	9	9.6	7	9.2	5	5.6	**35**
**May**	–	–	7	9.8	14	11.5	11	11.8	7	9.2	20	22.4	**59**
**June**	–	–	15	21.1	9	7.4	10	10.7	7	9.2	14	15.7	**55**
**July**	–	–	5	7.04	19	15.7	6	6.4	8	10.5	17	19.1	**55**
**August**	–	–	1	1.4	15	12.3	4	4.3	7	9.2	5	5.6	**32**
**September**	–	–	3	4.2	6	4.9	5	5.3	3	3.9	–	–	**17**
**October**	–	–	5	7.0	5	4.1	8	8.6	3	3.9	–	–	**21**
**November**	–	–	4	5.6	8	6.6	7	7.5	11	14.4	–	–	**30**
**December**	–	–	3	4.2	8	6.6	12	12.9	6	7.8	–	–	**29**
**Total**	**–**	**–**	**71**		**121**		**93**		**76**		**89**		**450**
