# Supplementary figures and images for: A data-based study in support of Blackbuck related cases from Haryana
Source: Data Brief. 2018 Feb 16;17:1196–200. doi: 10.1016/j.dib.2018.02.034 (PMC5966515; doi:10.1016/j.dib.2018.02.034)

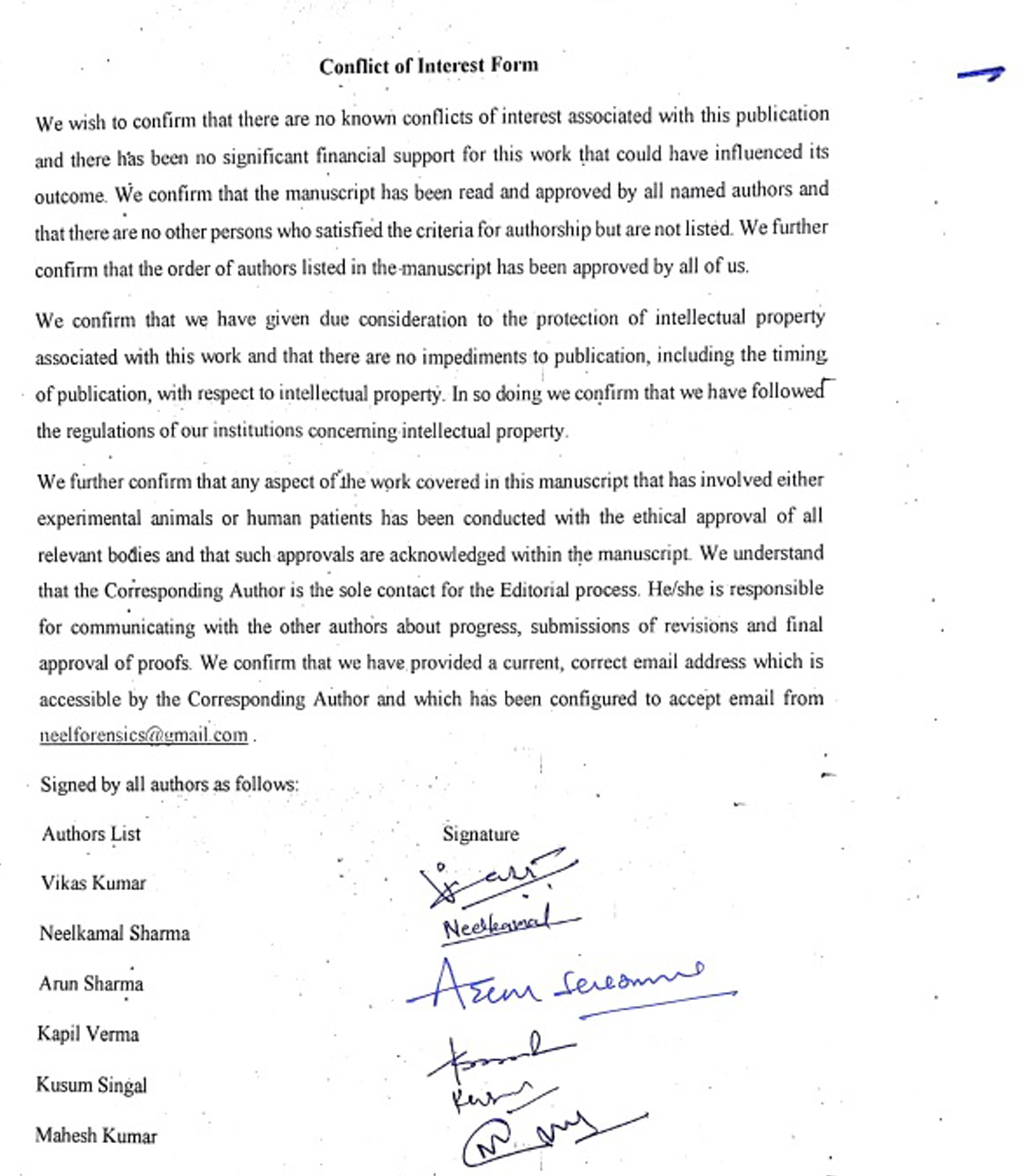

Supplement: Supplementary file 1 — Supplementary material [file mmc1.jpg]
